# Investigation of previously implicated genetic variants in chronic tic disorders: a transmission disequilibrium test approach

**DOI:** 10.1007/s00406-017-0808-8

**Published:** 2017-05-29

**Authors:** Mohamed Abdulkadir, Douglas Londono, Derek Gordon, Thomas V. Fernandez, Lawrence W. Brown, Keun-Ah Cheon, Barbara J. Coffey, Lonneke Elzerman, Carolin Fremer, Odette Fründt, Blanca Garcia-Delgar, Donald L. Gilbert, Dorothy E. Grice, Tammy Hedderly, Isobel Heyman, Hyun Ju Hong, Chaim Huyser, Laura Ibanez-Gomez, Ewgeni Jakubovski, Young Key Kim, Young Shin Kim, Yun-Joo Koh, Sodahm Kook, Samuel Kuperman, Bennett Leventhal, Andrea G. Ludolph, Marcos Madruga-Garrido, Athanasios Maras, Pablo Mir, Astrid Morer, Kirsten Müller-Vahl, Alexander Münchau, Tara L. Murphy, Kerstin J. Plessen, Veit Roessner, Eun-Young Shin, Dong-Ho Song, Jungeun Song, Jennifer Tübing, Els van den Ban, Frank Visscher, Sina Wanderer, Martin Woods, Samuel H. Zinner, Robert A. King, Jay A. Tischfield, Gary A. Heiman, Pieter J. Hoekstra, Andrea Dietrich

**Affiliations:** 1 Department of Genetics, Human Genetics Institute of New Jersey, Rutgers, The State University of New Jersey, Piscataway, NJ USA; 2 Department of Child and Adolescent Psychiatry, University Medical Center Groningen, University of Groningen, Groningen, The Netherlands; 3 Department of Psychiatry, Yale Child Study Center, Yale University School of Medicine, New Haven, CT USA; 4 Children’s Hospital of Philadelphia, Philadelphia, PA USA; 5 Yonsei University College of Medicine, Yonsei Yoo & Kim Mental Health Clinic, Seoul, South Korea; 6 Division of Tics, OCD and Related Disorders, Department of Psychiatry, Icahn School of Medicine at Mount Sinai, New York, NY USA; 7 Nathan S. Kline Institute for Psychiatric Research, Orangeburg, NY USA; 8 Yulius Academy and Division Child and Adolescent Psychiatry, Yulius Mental Health Organization, Barendrecht, The Netherlands; 9 Medizinische Hochschule Hannover Klinik für Psychiatrie, Sozialpsychiatrie und Psychotherapie, Hannover, Germany; 10 University Hospital Medical Center Hamburg-Eppendorf, Hamburg, Germany; 11 Department of Child and Adolescent Psychiatry and Psychology, Institute of Neurosciences, Hospital Clinic Universitari, Barcelona, Spain; 12 Cincinnati Children’s Hospital Medical Center, Cincinnati, OH USA; 13 Evelina London Children’s Hospital GSTT, Kings Health Partners AHSC, London, UK; 14 Great Ormond Street Hospital for Children, and UCL Institute of Child Health, London, UK; 15 Hallym University Sacred Heart Hospital, Anyang, South Korea; 16 De Bascule, Amsterdam, The Netherlands; 17 AMC Department of Child and Adolescent Psychiatry, Amsterdam, The Netherlands; 18 Yonsei Bom Clinic, Seoul, South Korea; 19 Department of Psychiatry, University of California, San Francisco, USA; 20 Korea Institute for Children’s Social Development, Seoul, South Korea; 21 Kangbuk Samsung Hospital, Seoul, South Korea; 22 University of Iowa Carver College of Medicine, Iowa City, IA USA; 23 Department of Child and Adolescent Psychiatry and Psychotherapy, University of Ulm, Ulm, Germany; 24 Sección de Neuropediatría, Instituto de Biomedicina de Sevilla, Hospital Universitario Virgen del Rocío/CSIC/Universidad de Sevilla, Seville, Spain; 25 Department of Child and Adolescent Psychiatry, Erasmus Medical Center-Sophia Children’s Hospital, Rotterdam, The Netherlands; 26 Unidad de Trastornos del Movimiento, Instituto de Biomedicina de Sevilla (IBiS), Hospital Universitario Virgen del Rocío/CSIC/Universidad de Sevilla, Seville, Spain; 27 Institut d’Investigacions Biomediques August Pi i Sunyer (IDIPABS) and Centro de Investigacion en Red de Salud Mental (CIBERSAM), Barcelona, Spain; 28 Institute of Neurogenetics, University of Lübeck, Lübeck, Germany; 29 Child and Adolescent Mental Health Center, Mental Health Services, Capital Region of Denmark and Faculty of Health Sciences, University of Copenhagen, Copenhagen, Denmark; 30 Department of Child and Adolescent Psychiatry, TU Dresden, Dresden, Germany; 31 National Health Insurance Service Ilsan Hospital, Goyang-Si, South Korea; 32 Youth Division, Altrecht, Institute for Mental Health, Utrecht, The Netherlands; 33 Department of Neurology, Admiraal De Ruyter Ziekenhuis, Goes, The Netherlands; 34 Department of Pediatrics, University of Washington, Seattle, WA USA

**Keywords:** Attention-deficit/hyperactivity disorder, Candidate gene study, Obsessive–compulsive disorder, Tourette syndrome, Transmission Disequilibrium Test

## Abstract

Genetic studies in Tourette syndrome (TS) are characterized by scattered and poorly replicated findings. We aimed to replicate findings from candidate gene and genome-wide association studies (GWAS). Our cohort included 465 probands with chronic tic disorder (93% TS) and both parents from 412 families (some probands were siblings). We assessed 75 single nucleotide polymorphisms (SNPs) in 465 parent–child trios; 117 additional SNPs in 211 trios; and 4 additional SNPs in 254 trios. We performed SNP and gene-based transmission disequilibrium tests and compared nominally significant SNP results with those from a large independent case–control cohort. After quality control 71 SNPs were available in 371 trios; 112 SNPs in 179 trios; and 3 SNPs in 192 trios. 17 were candidate SNPs implicated in TS and 2 were implicated in obsessive–compulsive disorder (OCD) or autism spectrum disorder (ASD); 142 were tagging SNPs from eight monoamine neurotransmitter-related genes (including dopamine and serotonin); 10 were top SNPs from TS GWAS; and 13 top SNPs from attention-deficit/hyperactivity disorder, OCD, or ASD GWAS. None of the SNPs or genes reached significance after adjustment for multiple testing. We observed nominal significance for the candidate SNPs rs3744161 (*TBCD*) and rs4565946 (*TPH2*) and for five tagging SNPs; none of these showed significance in the independent cohort. Also, *SLC1A1* in our gene-based analysis and two TS GWAS SNPs showed nominal significance, rs11603305 (intergenic) and rs621942 (*PICALM*). We found no convincing support for previously implicated genetic polymorphisms. Targeted re-sequencing should fully appreciate the relevance of candidate genes.

**Electronic supplementary material:**

The online version of this article (doi:10.1007/s00406-017-0808-8) contains supplementary material, which is available to authorized users.

## Introduction

Both family and twin studies have consistently suggested a genetic etiology of Tourette syndrome (TS), a common childhood-onset tic disorder [1]. The strong heritability has led to a wide range of gene finding efforts, which initially, prior to the initiation of genome-wide association studies (GWAS) focused on family-based linkage and candidate-gene-based case–control studies [1, 2]. These candidate genes have typically been selected based on prevailing theories of the etiology of TS. There has now been a considerable number of candidate genes studies which have attempted to confirm theories on neurotransmitter involvement in TS [2]. However, the field of candidate gene studies is characterized by poorly replicated findings using mostly small sample sizes [1, 2]. For example, while the *dopamine receptor D2* (*DRD2*) gene was implicated by Comings et al. [3] this finding could not be replicated in a subsequent study led by Díaz-Anzaldúa et al. [4]. Currently, we lack a comprehensive and independent synthesis of the various putative genetic loci identified from candidate gene studies.

Neurotransmitter-related candidate gene studies in TS have been based on post-mortem brain findings [5], therapeutic responses to antipsychotics [5], pathophysiological studies [5], or genetic linkage studies [2]; and have included genes related to the neurotransmitter pathways dopamine, glutamate, histamine, and serotonin [2, 5]. The classical principle guiding the investigation of candidate genes in these pathways has been the thought that certain single nucleotide polymorphisms (SNPs) within these genes might impact protein functions required for normal neurotransmission.

The one published GWAS study of TS to date [6] did not result in findings with genome-wide significance, possibly due to lack of power to detect common variants of small effects. A subsequent study [7] looking into the top SNPs was only able to find significance for a SNP (rs2060546) in the *Netrin 4* gene (*NTN4*), following correction for multiple statistical comparisons. However, this study [7] and others [8] have not replicated the original top TS GWAS signal (rs7868992) [6] in the *Collagen Type XXVII Alpha 1* gene (*COL27A1*).

The aim of the present study was to independently replicate findings of candidate SNPs and candidate genes previously implicated in TS or related disorders that are often comorbid with TS, i.e., obsessive–compulsive disorder (OCD), attention-deficit/hyperactivity disorder (ADHD), and autism spectrum disorder (ASD) [9], given a potential shared genetic susceptibility [2]. We investigated a total of 196 SNPs that included the following: individual candidate SNPs; tagging SNPs (tSNPs) covering neurotransmitter-related candidate genes; top SNPs from TS GWAS; and top SNPs from GWAS of related disorders. Analyses were performed as part of the Tourette International Collaborative Genetics (TIC Genetics, [10]) study, and consisted of 465 children with a chronic tic disorder and both parents. The selected genetic loci were investigated in relation to the presence of a chronic tic disorder in SNP and gene based transmission disequilibrium tests (TDT) analyses. The use of the TDT is a major advantage above a case–control design as it ensures proper control for population stratification with no need for a separate control group [11].

## Methods

### Study subjects

This study included 465 parent–child trios from 412 families (some parents formed trios with more than one affected child), with probands affected with a chronic tic disorder, of whom 93% had TS and 7% a chronic motor or vocal tic disorder. Probands (77.8% males; mean age = 13.9, SD = 6.42, range 4–52 years) and their biological parents were from the Tourette International Collaborative Genetics (TIC Genetics, [10]) study, recruited between 2011 and 2014 across 24 sites in the USA, Europe, and South Korea (360 parent–child trios); the New Jersey Center for Tourette Syndrome (NJCTS) [12] between 2006 and 2010 (102 parent–child trios); or the Yale Child Study Center in 2007 (three parent–child trios). The TIC Genetics study was established as a comprehensive gene discovery effort for TS, with a focus on multiply-affected family pedigrees and cases without a family history of tics. Inclusion criteria of cases were presence of a chronic tic disorder according to the Diagnostic and Statistical Manual of Mental Disorders Fourth edition, Text Revision (DSM-IV-TR, [13]) and donation of DNA by the proband and both biological parents. Before enrolling in the study, all adult participants and parents of children provided written informed consent along with written or oral assent of their participating child. The Institutional Review Board of each participating site had approved the study.

### Clinical measures

Experienced clinicians assigned a clinical diagnosis of a tic disorder and assessed the possible presence of comorbid OCD and/or ADHD based on DSM-IV-TR criteria, as described elsewhere in more detail [10].

### Selection of single nucleotide polymorphisms

Selection of TS candidate SNPs and genes was based on review articles [1, 2] and PubMed searches using the terms “Tourette”, “tics”, and “TS” in combination with the terms “candidate gene” or “association study”, of literature published until October 2014. We selected 196 SNPs, including: (a) 17 individual candidate SNPs previously reported to be at least nominally significantly (*P* value <0.05) associated with TS; (b) 2 individual candidate SNPs previously associated with OCD and ASD, respectively; (c) 148 tSNPs covering seven neurotransmitter-related candidate genes of which at least one polymorphism had previously been reported to be at least nominally significantly (*P* value <0.05) associated with either TS (*DRD2*, *HDC*, *MAO*-*A*, *SLC6A3/DAT1*, and *TPH2*, reviewed in [2]) or a related disorder (*SLC1A1* and *GABRA2*, associated with, respectively, OCD and ASD [14, 15]), in addition to *HRH3* (which has never been investigated in relation to TS, but was included based on the possible involvement of genes related to histamine [16, 17]); tSNP selection was restricted to common SNPs (minor allele frequency >0.05) and were selected using the HapMap CEU population as a reference and the Tagger algorithm implemented in Haploview [18, 19]. The R^2^ threshold for the tSNP selection was set at 0.8. To capture possible regulatory variants, we also included tSNPs 10 kb upstream and downstream of each gene (see supplementary Table S1). (d) 12 TS GWAS-based top SNPs, i.e., the top 5 LD-independent SNPs from the first GWAS of TS [6], 4 top SNPs from the Gilles de la Tourette Syndrome Genome-Wide Association Study Replication Initiative [7], and the top 3 SNPs from the first cross-disorder GWAS of TS and OCD [20]; and (e) 17 top SNPs from GWAS studies of OCD [21], ADHD [22, 23], and ASD [24, 25]. We did not include the previously implicated TS SNPs rs1894236 (*HDC*), rs1056534 (*TBCD*), rs25531 (*SLC6A4*), rs25532 (*SLC6A4*), nor tSNPs covering the possible TS candidate genes *DRD4*, *Arylacetamide Deacetylase* (*AADAC*) [26, 27], and *Glial Cell Derived Neurotrophic Factor* (*GDNF*) [27], nor 19 SNPs recently implicated in a meta-analysis of TS and ADHD [28].

### Genotyping

Genomic DNA was extracted from peripheral blood using standard protocols. Genotyping was either performed with the Illumina GoldenGate Genotyping Assay (San Diego, CA, USA) on a custom-made array containing 192 SNPs at the Genome Analysis Facility of the University Medical Center in Groningen, Netherlands (211 of the parent–child trios). The remaining 254 parent–child trios were genotyped with the Illumina HumanOmniExpressExome v1.2 BeadChip genotyping array at the Yale Center for Genomic Analysis, USA. From this array, we selected the same SNPs, as far as these were available, which was the case for 75 of the 192 SNPs; plus we selected 4 additional SNPs. This resulted in a total of 196 SNPs. See supplementary Table S2 for the sample sizes for the different SNP analyses. Processing of the raw intensity data and calling of the genotypes was performed with the Illumina GenomeStudio software (V2011.1). The PLINK input files needed for further analysis were generated using the PLINK Report Plug-in (v2.1.3) in the GenomeStudio Software.

### Quality control

Quality control of the data was performed with PLINK v1.07 [29] and carried out using the recommended parameters [30]. Individuals were excluded because of (1) discordant sex information (GoldenGate Genotyping *N* = 13, HumanOmniExpressExome *N* = 5); (2) low genotyping call rate, i.e., less than 90% (GoldenGate Genotyping *N* = 7, HumanOmniExpressExome *N* = 9); (3) Mendelian errors and samples with error rates exceeding 10% (GoldenGate Genotyping *N* = 12, HumanOmniExpressExome *N* = 8), and (4) strand issues after merging data from genotyping arrays (HumanOmniExpressExome *N* = 19). Note that removal of one parent with several affected children led to the loss of several trios, bringing the total of excluded parent–child trios after quality control check to *N* = 94.

Furthermore, six SNPs not conforming to Hardy–Weinberg equilibrium or with genotyping call rate less than 90% were excluded (GoldenGate Genotyping *N* = 5, HumanOmniExpressExome *N* = 1). Finally, after merging the SNPs from both arrays, the quality of the SNPs was assessed again and four more SNPs did not conform to Hardy–Weinberg equilibrium, reducing the number of SNPs to 186. Considering that all members of each trio were genotyped using the same platform, no further corrections were necessary to control for possible batch effects between the two genotyped subsets.

### Family-based association analysis

Family-based association analysis was carried out with the Parent-TDT option in PLINK that utilizes both the standard TDT and the parental discordance test to look for association with chronic tic disorders. Empirical significance levels were generated with PLINK using max(T) permutation methods with 10,000 permutations. Pointwise significance threshold was set at *α* = 0.05. To avoid type I errors, for the SNP-based tests, correction for multiple testing was conducted using the false discovery rate (FDR). For the gene-based analyses, all tSNPs belonging to the same gene were grouped together and were analyzed using the set-based tests in conjunction with the TDT option in PLINK. In an attempt to replicate findings of Mössner et al. [31], we also conducted a follow-up analysis of the *TPH2* haplotype (rs4570625-rs4565946) using the haplotype-based TDT option in PLINK. Empirical *P* values were calculated for each gene and correction for multiple testing was again done using the FDR method. Post hoc power analyses for our TDT approach were carried out with the snpPower function in the R-package Haplin v6.0.1.

To reduce the risk of type II errors, we attempted replication of our nominally significant (*P* value <0.05) SNPs against nominal significance of the case–control comparisons stemming from the TS GWAS performed by the Tourette Syndrome Association International Consortium for Genetics (TSAICG) including 1285 cases and 4964 ancestry-matched controls [6]. Given the large number of markers tested in a GWAS and accompanying stringent correction for multiple testing, GWAS studies contain a large number of SNPs with nominal significance that do not survive correction for multiple comparisons of which, however, true involvement cannot be ruled out. We argued that only SNPs that would be nominally significant in both cohorts would unequivocally point towards involvement in chronic tic disorder and then would suggest that correction for multiple testing had been too stringent. Study subjects did not overlap between TSAICG and TIC Genetics.

## Results

### Sample description

Of the original 465 parent–child trios (from 412 families) and 196 SNPs, a maximum of 371 parent–child trios (from 328 families; 92% European Caucasian, 6% Asian, and 2% Black/African American or American Indian) and 186 SNPs remained eligible for analysis following our quality control, as described earlier. Note that not all of the 186 SNPs were available for all of the families (see Table S2); 71 SNPs were available for all 371 parent–child trios, 112 additional SNPs in 179 trios, and 3 additional SNPs in 192 trios. For an overview of the investigated SNPs and details from reference studies, see Tables 1, 2, 3 and supplementary Tables S1–2. The final set of probands with a chronic tic disorder consisted of 291 males and 80 females between 4 and 45 years of age (mean age = 13.6, SD = 5.80). In addition to TS, 60% of the patients had OCD and 43% had ADHD.

**Table 1:** Overview of investigated individual candidate SNPs previously implicated in TS, OCD, or ASD after quality control check including findings of reference studies

SNP ID	MAF	Gene name	CHR	Gene function	Type of reference study	Sample size reference study	*χ* ^2^ statistic reference study	OR reference study	No. of parent–child trios after quality control in present study	Power in present study^a^	References
Implicated in TS											
rs1800497	0.18	*ANKK1/DRD2*	11	Serine/threonine kinase	Case–control	147 TS/314 controls	19.4		179		[3]
rs9357271^b^	0.24	*BTBD9*	6	Protein–protein interactions	Case–control	322 TS/290 controls	8.02		371		[32]
rs11264126^c^	0.49	*DLGAP3*	1	Post-synaptic scaffolding protein	TDT	289 parent–child trios		1.41	371	0.91	[33]
rs12141243	0.11	*DLGAP3*	1	Post-synaptic scaffolding protein	TDT	289 parent–child trios		1.04	179	<0.10	[33]
rs6279	0.31	*DRD2*	11	Dopamine receptor	TDT	69 parent–child trios	11.5		179		[34]
rs1079597	0.11	*DRD2*	11	Dopamine receptor	TDT	69 parent–child trios	11.5		371		[34]
rs4648318	0.25	*DRD2*	11	Dopamine receptor	TDT	69 parent–child trios	11.5		371		[34]
rs854150	0.36	*HDC*	15	Histamine synthesis	TDT	520 TS families	8.13		179		[35]
rs518147	0.37	*HTR2C*	X	Serotonin receptor	Case–control	87 TS/311 controls		2.50	179	0.99	[36]
rs3813929	0.18	*HTR2C*	X	Serotonin receptor	Case–control	87 TS/311 controls		1.89	371	0.99	[36]
rs6347	0.24	*SLC6A3/DAT1*	5	Dopamine transporter	Case–control	266 cases/236 controls	8.40	1.78^d^	179	0.92	[37]
rs9593835^e^	0.26	*SLITRK1*	13	Neurite outgrowth	TDT	154 TS families	6.22	1.66^f^	371	0.99	[38]
rs9531520	0.19	*SLITRK1*	13	Neurite outgrowth	TDT	154 TS families	6.22	1.45^f^	179	0.55	[38]
rs3744161	0.46	*TBCD*	17	Tubulin folding protein	TDT	100 TS families			179		[39]
rs662669	0.42	*TBCD*	17	Tubulin folding protein	TDT	100 TS families			179		[39]
rs4565946	0.49	*TPH2*	12	Serotonin synthesis	Case–control	98 TS/178 controls		1.95/1.65^g^	371	0.99/0.99^g^	[31]
rs4570625	0.2	*TPH2*	12	Serotonin synthesis	Case–control	98 TS/178 controls		1.95/1.65^g^	371	0.99/0.98^g^	[31]
Implicated in related disorders											
rs4680	0.47	*COMT*	22	Dopamine degradation	Meta-analysis	47,358 cases OCD/68,942 controls/2433 OCD parent–child trios			371		[40]
rs7794745	0.31	*CNTNAP2*	7	Cell adhesion molecule	TDT	145 autism parent–child trios and 78 sib-pairs			371		[41]

All SNPs passed standard quality control checks in PLINK V1.07 using the recommended parameters published in [29, 30]

TS, Tourette syndrome; OCD, obsessive–compulsive disorder; ASD, autism spectrum disorder; MAF, minor allele frequency (based on the HapMap-CEU population); CHR, chromosome; OR, odds ratio; *ANKK1*, ankyrin repeat and kinase domain containing 1; *DRD2*, dopamine receptor D2; *BTBD9*, BTB (POZ) domain containing 9; *CNTNAP2*, contactin associated protein-like 2; *DLGAP3*, Discs, large (Drosophila) homolog-associated protein 3; TDT, transmission disequilibrium test; *HDC*, l-histidine decarboxylase; 5-HT receptor 2C; *SLC6A3*, solute carrier family 6, dopamine transporter; *DAT1*, dopamine transporter 1; *SLITRK1*, SLIT and NTRK-like family, Member 1; *TBCD*, tubulin folding cofactor; *TPH2*, tryptophan hydroxylase 2; *COMT*, catechol-O-methyltransferase

^a^When the reference study had provided an odds ratio, we calculated the power in the present study, based on the number of available parent–child trios for each SNP (Table S2), the reported minor allele frequency, odds ratio in the reference study, and *α* = 0.05 (see also Table S3)

^b^This SNP is in high LD (*R* ^2^ > 0.8) with the implicated SNPs rs4714156 and rs9296249

^c^rs11264126 was nominally significant and together with rs12141243 nominally significant in two haplotypes

^d^Odds ratio reported for the genotypic comparison of the AG versus the AA genotype

^e^This SNP is in high LD (*R* ^2^ > 0.8) with the implicated SNP rs9546538

^f^Odds ratios were obtained from the single marker analysis

^g^Results reported of the haplotype-based analysis of two haplotypes containing rs4565946 and rs457062

**Table 2:** Overview of investigated candidate genes previously implicated in TS, OCD, or ASD

Gene	# of tSNPs	# of tSNPs excluded following QC^a^	CHR	Neurotransmitter pathway	Function	Sample size reference study	References
TS neurotransmitter-related candidate genes							
* DRD2*	14		11	Dopamine	Dopamine receptor	147 TS/314 controls	[3]
* HDC*	*11*		15	Histamine	Histamine synthesis	520 TS families	[17]
* MAO*-*A*	*9*	1	X	Serotonin (5-HT), dopamine	Degradation of dopamine and 5-HT	110 TS parent–child trios	[4]
* SLC6A3/DAT1*	*21*	3	5	Dopamine	Dopamine transporter	266 cases/236 controls	[37]
* TPH2*	*19*		12	Serotonin (5-HT)	5-HT synthesis	149 TS/125 controls	[42]
Candidate genes implicated in related disorders							
*GABRA2*	14		4	GABA	GABA receptor	470 autism families	[14]
*SLC1A1*	52		9	Glutamate	Glutamate transporter	377 OCD families	[15]
Newly investigated candidate gene							
*HRH3*	8	2	20	Histamine	Histamine receptor		

TS, Tourette syndrome; OCD, obsessive–compulsive disorder; ASD, autism spectrum disorder; tSNPs, tagging SNPs; QC, quality check; CHR, chromosome; *DRD2*, dopamine receptor D2; *HDC*, l-histidine decarboxylase; *MAO*-*A*, monoamine oxidase-A; *SLC6A3/DAT1*, solute carrier family 6/dopamine transporter; *TPH2*, tryptophan hydroxylase 2; *GABRA2*, GABA-A receptor, alpha 2; *SLC1A1*, solute carrier family 1 member 1, glutamate transporter; *HRH3*, histamine receptor H3

^a^SNPs were excluded following standard quality control checks in PLINK V1.07 using the recommended parameters published in [29, 30]

**Table 3:** Overview of investigated top SNPs implicated in GWAS of TS, OCD, ADHD, or ASD

*SNP*	MAF	Gene name	CHR	Function	Sample size reference study	No. of parent–child trios after quality control in present study	References
TS GWAS SNPs							
rs7868992	0.28	*COL27A1*	9	Calcification of cartilage and the transition of cartilage to bone	1285 cases/4964 controls	371	[6]
rs621942	0.24	*PICALM*	11	Endocytosis	1894 cases/5574 controls	192	[7]
rs6539267	0.27	*POLR3B*	12	DNA-dependent RNA polymerase	1285 cases/4964 controls	371	[6]
rs4988462	0.44	*POU1F1*	3	Transcription factor	2723 cases/5667 controls	179	[20]
rs7123010	0.27	*ME3*	11	Malate metabolism	1894 cases/5574 controls		[7]
rs2060546^a^	0.01		12		1894 cases/5574 controls		[7]
rs13063502	0.18		3		1285 cases/4964 controls	371	[6]
rs769111	0.37		7		1285 cases/4964 controls	179	[6]
rs7336083	0.33		13		1285 cases/4964 controls	371	[6]
rs11603305	0.32		11		1894 cases/5574 controls	192	[7]
rs11149058	0.22		13		2723 cases/5667 controls	179	[20]
rs4271390^a^	0.22		11		2723 cases/5667 controls		[20]
OCD GWAS SNPs							
rs11081062	0.19	*DLGAP1*	18	Scaffold protein	1465 cases/5557 controls/400 parent–child trios	371	[21]
rs9499708	0.42		6		1465 cases/5557 controls/400 parent–child trios	179	[21]
rs9652236	0.15		13		1465 cases/5557 controls/400 parent–child trios	371	[21]
rs6131295	0.23		20		1465 cases/5557 controls/400 parent–child trios	371	[21]
rs297941^a^	0.44		12		1465 cases/5557 controls/400 parent–child trios		[21]
ADHD GWAS SNPs							
rs2556378	0.18	*BCL11A*	2	Myeloid and B-cell proto-oncogene	495 cases/1300 controls	179	[22]
rs12575642	0.15	*FERMT3*	11	Cell adhesion	465 trios	371	[23]
rs5016282	0.15	*GRM5*	11	Glutamate receptor	495 cases/1300 controls	179	[22]
rs12037173	0.07	*LRRC7*	1	Cell adhesion, dendritic branching, and neuronal excitability	465 parent–child trios	179	[23]
rs11607165	0.15	*STIP1*	11	Response to stress	465 parent–child trios	371	[23]
ASD GWAS SNPs							
rs1718101	0.07	*CNTNAP2*	7	Cell adhesion	2705 families	179	[24]
rs4675502	0.37	*PARD3B*	2	Cell division and cell polarization	2705 families	179	[24]
rs4150167^a^	0.04	*TAF1C*	16	Transcription factor	2705 families		[24]
rs4307059	0.37		5		780 families/1204 cases/6491 cases	179	[25]
rs13176113^b^	0.28		5		780 families/1204 cases/6491 cases	179	[25]
rs7834018	0.10		8		2705 families	371	[24]
rs7711337	0.40		5		2705 families	371	[24]

TS, Tourette syndrome; GWAS, genome-wide association study; OCD, obsessive–compulsive disorder; ADHD, attention-deficit/hyperactivity disorder; ASD, autism spectrum disorder; MAF, minor allele frequency (based on 1000 genomes); CHR, chromosome; *COL27A1*, *Collagen, Type XXVII, Alpha 1*; *PICALM*, *Phosphatidylinositol Binding Clathrin Assembly Protein*; *POLR3B*, P*olymerase (RNA) III (DNA Directed) Polypeptide B*; *POU1F1*, *POU Class 1 Homeobox 1*; *ME3*, *Malic Enzyme 3*; *DLGAP1*, *Discs, Large (Drosophila) Homolog*-*Associated Protein 1*; *BCL11A*, *B*-*Cell CLL/Lymphoma 11A*; *FERMT3*, *Fermitin Family Member 3*; *GRM5*, *Glutamate Receptor, Metabotropic 5*; *LRRC7*, *Leucine Rich Repeat Containing 7*; *STIP1*, *Stress*-*Induced Phosphoprotein 1*; *CNTNAP2*, *Contactin Associated Protein*-*Like 2*; *PARD3B*, *Par*-*3 Family Cell Polarity Regulator Beta*; *TAF1C*, *TATA Box Binding Protein (TBP)*-*Associated Factor*

^a^SNP did not pass standard quality control checks in PLINK V1.07 using the recommended parameters published in [29, 30]

^b^Original GWAS reported results for rs7704909 that is in high LD (*R* ^2^ = 1) with rs13176113

### Transmission disequilibrium tests

#### Candidate SNPs previously implicated in TS

None of the SNPs’ p values passed the FDR threshold taking multiple testing into account. However, at nominal significance (*P* value < 0.05), the TDT revealed over-transmission of the minor alleles of rs3744161 and rs4565946 located in the *TBCD* and *TPH2* gene, respectively (Table 4). A follow-up analysis on the *TPH2* haplotype (rs4570625-rs4565946) showed no significant association with chronic tic disorders (Table S4). For the majority of the investigated SNPs, we did not find any indication for involvement at the level of nominal significance (Table S5). We did not replicate the previously implicated *SLITRK1* SNPs rs9593835 and rs9531520 [38].

**Table 4:** Nominally significant SNP findings from our transmission disequilibrium tests with corresponding *P* values from the TS GWAS of the Tourette Syndrome Association International Consortium for Genetics of the previously implicated TS candidate SNPs or tSNPS from previously implicated candidate genes

SNP	CHR	BP	Gene	Minor/major allele	MAF	Over-transmitted allele	T:U	OR^a^	95% CI^a^	*χ* ^2 b^	*P* value nominal^c^	*P* value adjusted (FDR)^d^	OR TS GWAS^e^	*P* value TS GWAS^e^
Nominally significant candidate SNPs previously implicated in TS														
rs3744161	17	80828057	*TBCD*	G/C	0.49	Minor	123:91	1.35	1.03–1.77	5.45	0.03	0.64	0.97	0.31
rs4565946	12	72336769	*TPH2*	T/C	0.49	Minor	205:163	1.26	1.02–1.55	6.01	0.02	0.57	1.01	0.81
Nominally significant tSNPs from candidate genes previously implicated in TS or OCD														
rs17812372	9	4519989	*SLC1A1*	G/C	0.04	Minor	49:24	2.04	1.25–3.33	11.23	0.0006	0.11	1.06	0.29
rs1042098^f^	5	1394815	*SLC6A3/DAT1*	G/A	0.29	Minor	159:118	1.35	1.06–1.71	4.96	0.03	0.71	1.00	0.98
rs11615016^f^	12	72415994	*TPH2*	G/A	0.03	Minor	42:28	1.5	0.93–2.42	7.35	0.006	0.57	0.97	0.63
rs4760813^f^	12	72322894	*TPH2*	C/G	0.30	Major	58:39	1.49	0.99–2.23	4.24	0.04	0.71	1.01	0.85
rs7969998^f^	12	72328745	*TPH2*	C/T	0.05	Major	57:41	1.39	0.93–2.08	7.38	0.01	0.57	0.95	0.29
Nominally significant SNPs from TS GWAS studies														
rs11603305	11	10997949	Intergenic	G/A	0.32	Minor	125:92	1.36	1.04–1.78	6.11	0.02	0.24		
rs621942	11	85783738	*PICALM*	A/C	0.24	Minor	121:84	1.44	1.09–1.90	7.08	0.01	0.24		

SNP, single nucleotide polymorphism; CHR, chromosome; BP, base pair position (Build GRCh37); MAF, minor allele frequency (based on 1000 genomes); T:U, transmitted:untransmitted count; OR, odds ratio; FDR; false discovery rate; SLC1A1, *solute carrier family 1 member 1, glutamate transporter*; *SLC6A3*, *solute carrier family 6, dopamine transporter*; *DAT1*, *Dopamine Transporter 1*; *TBCD*, *Tubulin Folding Cofactor*; *TPH2*, *tryptophan hydroxylase 2*

^a^The odds ratios and 95% confidence intervals presented are based on the standard transmission disequilibrium test in PLINK

^b^The *χ* ^2^ test statistic is derived from the Parent-TDT option in plink

^c^Empirical *P* value for the gene based on 10,000 permutations

^d^ *P* value adjusted for multiple comparisons using the FDR for all SNPs that passed quality control checks

^e^Based on case–control comparisons from the TSAICG cohort [6]. Note that uncorrected *P* values are reported

^f^The gene-based analysis showed no evidence of association, however, this particular SNP did show nominal significance when separately analyzed

#### Candidate genes previously implicated in TS or related disorders

Similarly, for our gene-based analyses, none of our findings met the threshold for statistical significance, adjusted for multiple testing. We only found a nominally significant association for the *glutamate transporter* gene *SLC1A1* with chronic tic disorder (*P* value = 0.02, Table S6). In addition, a number of individual tSNPs from the candidate genes reached nominal significance (Table 4). *SNPs previously implicated in GWAS of TS and related disorders.* None of these met the FDR threshold (Table S7). We found nominal significance for two top TS GWAS SNPs (Table 4), i.e., one intergenic SNP variant (rs11603305) and rs621942 of the *PICALM* gene [6, 7].

### Comparison of nominally significant SNPs with independent cohort

None of our nominally significant SNPs, including the previously implicated candidate SNPs and the individual tSNPs from the candidate genes, showed a nominally significant odds ratio in the TSAICG cohort [6] (Table 4). Note that we did not compare our two nominally significant TS GWAS SNPs (rs11603305 and rs621942) as they were derived from the TSAICG cohort.

### Post hoc power analyses

For those 75 SNP analyses for which we had our maximum available sample size of 371 parent–child trios, our study was sufficiently powered (power ≥80%) to detect an odds ratio of 1.8 for rare SNPs (MAF = 0.05) and an odds ratio of 1.4 for more common SNPs (MAF ≥ 0.20; see further Table 1 and Table S3), while for those SNP analyses that were only genotyped in a subset of the trios (for most of the SNPs, *N* = 179) our study was sufficiently powered (power ≥80%) to detect common SNPs (MAF ≥ 0.20) with an odds ratio of 1.6 or more (Table S3), and an *α* = 0.05. For all of the previously implicated candidate SNPs we did obtain the desired power of 80% except for rs12141243 (*DLGAP3*) and rs9531520 (*SLITRK1*) (Table 1).

## Discussion

The goal of this study was to provide a synthesis of previously implicated candidate SNPs, candidate genes, and top SNPs from recent GWAS of TS and related disorders. Following correction for multiple testing, we did not find evidence for involvement for the previously implicated neurotransmitter-related candidate genes (*DRD2*, *HDC*, *MAO*-*A*, *SLC6A3*/*DAT1*, *TPH2*, *COMT*, *GABRA2*, *SLC1A1*, and *HRH3*), SNPs previously implicated in candidate genes (*BTBD9, CNTNAP2*, *DLGAP3*, *SLITRK1*, and *TBCD*), and top SNPs from GWAS of TS and related disorders. We also did not find evidence for the top five LD-independent SNPs from the first GWAS of TS [6] and the *SLITRK1* candidate gene [38]. This non-replication of candidate genes is in line with findings in other neuropsychiatric disorders [43, 44].

Both pharmacological evidence and neuroimaging studies have pointed towards involvement of the dopamine pathway, and based on these findings several groups have investigated genes within this pathway, mostly with inconsistent results [2]. Included in our study are the *dopamine receptor D2* (*DRD2*) and the *dopamine transporter* (*SLC6A3*/*DAT1*) gene that were both implicated in TS by others [3, 34, 37] and the *catechol*-*O*-*methyltransferase* (*COMT*) gene that was implicated in OCD [45], a related disorder. Our findings for the *DRD2* gene are in contrast with the findings of Herzberg et al. and Comings et al. [3, 34] as both our investigation of previously implicated SNPs (rs1800497, rs6279, rs1079597, and rs4648318) and our analysis of the entire gene yielded no significant association. The differences in findings could be due to our increased sample size, as both previous studies included less than 150 cases [3, 34]. Similarly, we did not find evidence for *SLC6A3*, as both our analysis of a previously implicated SNP (rs6347) [37] and our analysis of the entire gene showed no association with chronic tic disorder. This discrepancy might be explained by the use of different analytical approaches, as Yoon et al. employed a case–control analysis. Finally, we found no evidence for the *COMT* SNP rs4680; however, this gene has never been associated with chronic tic disorders before but is strongly implicated in OCD [45].

Serotonin is another well-studied neurotransmitter pathway. Studies have shown a reduced concentration of serotonin and its metabolite in the brain and cerebrospinal fluid of TS patients [5]. Included in our study were SNPs in genes belonging to the serotonin receptor *HTR2C*, monoamine oxidase-A (*MAO*-A), and the tryptophan hydroxylase 2 (*TPH2*) gene, of which the latter is responsible for the synthesis of serotonin in the brain [31]. In contrast to the findings of Dehning et al. [36], we found no evidence for the *HTR2C* SNPs rs3813929 and rs518147. With regard to *THP2*, both previously implicated SNPs (rs4565946 and rs4570625) [31, 42] showed no evidence for association, although rs4565946 indicated a weak nominally significant signal that did not pass the threshold for significance when corrected for multiple testing. Further investigation of the *THP2* gene in our gene-based analysis and haplotype-based analysis of the haplotype rs4570625-rs4565946 showed no evidence for association. *MAO*-*A* is a well-known neurotransmitter gene that is responsible for both the degradation of serotonin and dopamine [2] and while the *MAO*-*A* promoter variable number of tandem repeats polymorphism was previously implicated in TS by Díaz-Anzaldúa et al. [4], *MAO*-*A* SNPs were not implicated in our study.

Following the finding of Ercan-Sencicek et al. of a rare mutation in the *Histidine Decarboxylase* (*HDC*) gene in a TS family, the histamine pathway has garnered much interest [17, 35, 46]. *HDC* encodes for a gene necessary for the synthesis of histamine, that functions as a neurotransmitter but is also involved in gastric acid secretion, immune system, bronchoconstriction, and vasodilation [17, 35]. However, we did not find a significant association for the *HDC* candidate gene or the previously implicated *HDC* SNP rs854150; this is in contrast with several studies [17, 35, 46], but is consistent with the finding of others [6, 47, 48]. We further investigated the histamine pathway by investigating another pathway gene that was not previously investigated in relation to chronic tic disorders: the *histamine receptor H3* (*HRH3*) gene. Here, we also found no association between this gene and chronic tic disorders. Considering that the initial *HDC* mutation is extremely rare [17] and that TS is considered a heterogeneous disorder [2], it is therefore likely that variants in the *HDC* gene, or in a broader sense variants in the histamine pathway, only cause tics in a subset of chronic tic cases.

Glutamate and gamma-aminobutyric acid (GABA) are major neurotransmitter pathways that may play a role in TS [5]. Glutamate and GABA play opposing roles as important excitatory and inhibitory neurotransmitter pathways in the central nervous system, respectively [2]. We did not find associations between chronic tic disorders and the *glutamate transporter* (*SLC1A1*) gene that has been implicated in OCD [15], or the *GABA*-*A receptor, alpha 2* (*GABRA2*) gene that has been implicated in autism [14].

Moving away from neurotransmitter pathways, there is a growing body of literature [2] implicating SNPs in candidate genes with a more structural function such as: the *BTB domain containing 9* (*BTBD9*), *contactin associated protein*-*like 2* (*CNTNAP2*), *discs large homolog-associated protein 3* (*DLGAP3), SLIT and NTRK-like family member 1* (*SLITRK1*), and the *tubulin folding cofactor D* (*TBCD*) gene [39]. We found no evidence for an association between SNPs in these genes and chronic tic disorders. *SLITRK1* is the most-studied gene and is functionally involved in neurite outgrowth [2]. We were unable to replicate the *SLITRK1* SNPs rs9593835 and rs9531520 which is in line with most TS studies [49–53], but not others [38, 54, 55]. Because of the inconsistent results in the past, there is an ongoing discussion whether de novo or transmitted *SLITRK1* variants contribute to TS [52]. Our findings do not support an association.

Further, our study was unable to demonstrate associations between chronic tic disorders and previously implicated SNPs from GWAS of TS, OCD, ADHD, and ASD [6, 7, 20–25]. Particularly, we found no associations for the top five LD-independent SNPs from the first GWAS of TS [6], including the top signal (rs7868992). Unfortunately, one of the top GWAS SNPs (rs2060546) did not pass standard quality controls checks, an SNP closest to *NTN4*, an axon guidance molecule expressed in developing striatum that was recently replicated by Paschou et al. [7].

A strength of our study is the well-characterized sample of parent–child trios. Use of TDT analysis eliminated population stratification bias, a major advantage over classical case–control studies [11]. Our post hoc power analyses demonstrated that, based on reported effect sizes, our study was sufficiently powered to detect associations for most of the previously implicated TS candidate SNPs. However, this was not the case for one of the candidate SNPs from *SLITRK1* and one from *DLGAP3*. As another strength, we used the large TSAICG case–control study [6] as a comparison sample of our nominally significant findings. A limitation of TDT is that only the heterozygous parents are informative. SNP loci that are less polymorphic are not optimally studied by this method. Importantly, it should also be noted that our study focused solely on SNPs rather than rare copy number variations (CNVs) or repeat polymorphisms. Thus, non-significant genes such as *MAO*-*A* and *COMT* may still play a role in TS through these other variant types [16, 56]. Our study also does not rule out that the investigated genes could still be involved in gene–gene interactions and gene–environment interactions or through rare mutations that can only be revealed through targeted re-sequencing [57]. For example, Alexander et al. found four deleterious mutations in the *SLITRK1* gene and one deleterious mutation in the *HDC* gene [57]. Finally, while we attempted to include as many candidate genes and SNPs available with promising evidence, we are aware that our selection does not include every single SNP implicated by previous studies. However, we believe that our selection is a good representation of the most important candidate genes and SNPs in the TS literature, as reviewed in [2].

In conclusion, following corrections for multiple testing, our TDT study did not show statistically significant associations between chronic tic disorders and previously implicated SNPs and tSNPs within neurotransmitter-related candidate genes. Moreover, our nominally significant findings were not replicated in an independent cohort. This highlights the importance of exceptional caution in interpreting results from previous SNP-based candidate gene studies. The efforts in discovering genetic loci involved in TS etiology are comparable to other neuropsychiatric disorders where candidate gene studies have also shown non-replication across studies [43, 44]. Similar to conditions such as ASD [14], the genetic architecture of TS likely involves complex and heterogeneous inheritance of both common and rare variants in many different genes and biological pathways. Genome-wide studies of large cohorts that capture all of these types of variation and targeted re-sequencing efforts to detect rare mutations (also addressing candidate genes) could be better suited for studying the complex neurobiology of TS and chronic tic disorders. Also the use of polygenic risk scores could further enhance understanding the relevance of common TS-related SNPs [20]. Meta-analytic studies are currently underway that may further clarify or rule out the possible involvement of the candidate genes *TBCD, TPH2, SLC1A1,* and *SLC6A3,* and SNPs from GWAS studies, i.e., the intergenic SNP variant rs11603305 and rs621942 of the *PICALM* gene which were all nominally significant in our study.

## Electronic supplementary material

Below is the link to the electronic supplementary material.

Supplementary material 1 (DOCX 153 kb)
