# Supplementary material for: Investigation of previously implicated genetic variants in chronic tic disorders: a transmission disequilibrium test approach
Source: Eur Arch Psychiatry Clin Neurosci. 2017 May 29;268(3):301–16. doi: 10.1007/s00406-017-0808-8 (PMC5708161; doi:10.1007/s00406-017-0808-8)
Supplement: Supplementary file 1 — Supplementary material 1 (DOCX 153 kb) [file 406_2017_808_MOESM1_ESM.docx]

Table S1: Tagging SNP information of included candidate genes implicated in TS, OCD, and ASD^a^

| **TS neurotransmitter-related candidate genes** | **Neurotransmitter pathway** | **Selected tSNP** **^b^** | **Proxy SNP(s)** **^c^** |
| --- | --- | --- | --- |
| *DRD2* | Dopamine | rs2587548 | rs7122246, rs2734831, rs4587762, rs2734838, rs2587548, rs7131440, rs2734839, rs1076563, rs1107162, rs1116313, |
|  |  |  | rs4938017, rs2734837, rs2734833, rs12800853, rs12363125, rs12364051 |
|  |  | rs2587550 | rs1124493, rs2242592, rs1003641, rs2734841, rs6275, rs2734842, rs6276, rs6279, rs2587550 |
|  |  | rs7122454 | rs11214611, rs4648317, rs7122454, rs4350392, rs10891551, rs4938019 |
|  |  | rs4245148 | rs4936270, rs4245148, rs4936274, rs4581480, rs4460839, rs11214613 |
|  |  | rs17115583 | rs1124492, rs17115583, rs4436578, rs7125415, rs4620755 |
|  |  | rs4245147 | rs4245147, rs4274224, rs4936271, rs4245146 |
|  |  | rs2234689 | rs2734848, rs2234689 |
|  |  | rs12574471 | rs2440390, rs12574471 |
|  |  | rs12364283 | rs12364283 |
|  |  | rs12422191 | rs12422191 |
|  |  | rs10891556 | rs10891556 |
|  |  | rs11214606 | rs11214606 |
|  |  | rs7131056 | rs7131056 |
|  |  | rs17529477 | rs17529477 |
| *HDC* | Histamine | rs8029462 | rs10519263, rs8030902, rs7162842, rs13329426, rs7163191, rs13329494, rs16963464, rs1802536, rs13329489, rs8029462 |
|  |  | rs11856059 | rs11856059, rs17796788, rs10518836, rs17740669, rs17740607 |
|  |  | rs1365503 | rs10220755, rs2187576, rs2114447, rs1365503 |
|  |  | rs854157 | rs860526, rs854160, rs854157 |
|  |  | rs2853766 | rs2853766, rs1677254 |
|  |  | rs854158 | rs2238292, rs854158 |
|  |  | rs854159 | rs854159, rs854163 |
|  |  | rs3959666 | rs3959666 |
|  |  | rs2070595 | rs2070595 |
|  |  | rs8034597 | rs8034597 |
|  |  | rs9920021 | rs9920021 |
| *MAO-A* | Serotonin, dopamine | rs5906729 | rs5906729 |
|  |  | rs5905859 | rs5905859 |
|  |  | rs3027409 | rs3027409 |
|  |  | rs3027415 ^d^ | rs3027415 |
|  |  | rs3027399 | rs3027399 |
|  |  | rs6609257 | rs6609257 |
|  |  | rs12843533 | rs12843533 |
|  |  | rs1181275 | rs1181275 |
|  |  | rs5906957 | rs5906957 |
| *SLC6A3/DAT1* | Dopamine | rs458860 | rs460700, rs460000, rs393795, rs464528, rs458860, rs456082, rs463379, rs464061, rs409588, rs456774, rs250682 |
|  |  | rs250686 ^d^ | rs250681, rs37020, rs250686, rs420422, rs458334, rs464049 |
|  |  | rs11564772 | rs11564771, rs11564772 |
|  |  | rs11564758 | rs4975646, rs13161905, rs11737901, rs11564758, rs1048953 |
|  |  | rs10052016 | rs10040882, rs2042449, rs10052016, rs10053602 |
|  |  | rs1042098 | rs1042098, rs3863145 |
|  |  | rs2550948 | rs2550948, rs2937639 |
|  |  | rs12654851 | rs12654851, rs3756450 |
|  |  | rs27048 | rs27048 |
|  |  | rs6350 | rs6350 |
|  |  | rs2550956 | rs2550956 |
|  |  | rs37022 | rs37022 |
|  |  | rs2617605 ^d^ | rs2617605 |
|  |  | rs2735917 | rs2735917 |
|  |  | rs40184 | rs40184 |
|  |  | rs27074 | rs27074 |
|  |  | rs6869645 | rs11564767, rs6876225, rs6869645, rs11564764, rs11564765 |
|  |  | rs466630 ^d^ | rs466630 |
|  |  | rs10064525 | rs10064525 |
|  |  | rs246993 | rs4975636, rs246993 |
|  |  | rs12516758 | rs12516758 |
| *TPH2* | Serotonin | rs7955501 | rs10879345, rs7963720, rs3903502, rs1843811, rs7955501, rs4760755, rs4760754, rs9325202, rs6582073, rs10879352, |
|  |  |  | rs10879351, rs7305115, rs4641528, rs12322900, rs4760817, rs2171363, rs7979770, rs6582077, rs1386498, rs7967686, |
|  |  |  | rs11179039, rs4760750, rs7309686, rs2368049, rs10879346, rs10748190, rs1352250, rs6582078, rs4760816, rs7969114 |
|  |  | rs1386497 | rs1386494, rs1843809, rs10784944, rs7954568, rs7968346, rs4760814, rs4760749, rs1473473, rs4760818, rs1386496, |
|  |  |  | rs10784942, rs1487276, rs1843812, rs2046582, rs7299582, rs7963226, rs1007023, rs2367899, rs7300490, rs1386497, |
|  |  |  | rs990234, rs1386489, rs1386490, rs1487281 |
|  |  | rs17110536 | rs11179033, rs10506645, rs17110489, rs1487278, rs17110532, rs17110540, rs11179002, rs17110536, rs1023990, |
|  |  |  | rs11179023, rs11834114, rs1386491, rs17110690, rs10506646, rs17110627, rs12319219, rs11179027, rs10879350, |
|  |  |  | rs11834097, rs12229394, rs17110477 |
|  |  | rs10879358 | rs1386483, rs11179049, rs1386487, rs10879355, rs4430554, rs4474484, rs4488237, rs10879358, rs11179052, |
|  |  |  | rs1386482, rs4469933, rs2200579, rs1386486, rs1386485, rs11179050, rs4290270, rs11179059 |
|  |  | rs11178999 | rs2129575, rs11179000, rs4570625, rs6582071, rs11178999 |
|  |  | rs10748185 | rs10748185, rs4565946, rs4448731, rs10748186 |
|  |  | rs1386488 | rs7978482, rs7300641, rs1843810, rs1386493, rs6582072, rs1386488, rs1386492 |
|  |  | rs12231341 | rs12231356, rs11179056, rs12231341 |
|  |  | rs4760820 | rs4760820 |
|  |  | rs17722134 | rs17722134 |
|  |  | rs4760813 | rs4760813 |
|  |  | rs17110747 | rs17110747 |
|  |  | rs1872824 | rs1872824 |
|  |  | rs11178993 | rs11178993 |
|  |  | rs11615016 | rs11615016 |
|  |  | rs10879357 | rs10879357 |
|  |  | rs1487275 | rs1487275 |
|  |  | rs7969998 | rs7969998 |
|  |  | rs12424836 | rs12424836 |
| **Comorbidity related candidate genes** |  |  |  |
| *GABRA2* | GABA | rs10013922 | rs1442061, rs10013922, rs1442062, rs9291283, rs13152740 |
|  |  | rs279828 | rs183960, rs279846, rs190715, rs279844, rs279831, rs279845, rs426463, rs279833, rs279828, rs12647055, rs175929, |
|  |  |  | rs10805145, rs279826, rs189957, rs4540087 |
|  |  | rs1372472 | rs2119767, rs1372472 |
|  |  | rs17537141 | rs3775282, rs17537141 |
|  |  | rs16859227 | rs3822051, rs16859227 |
|  |  | rs1442060 | rs1442060 |
|  |  | rs16859354 | rs16859354 |
|  |  | rs3849591 | rs3849591 |
|  |  | rs17537359 | rs17537359 |
|  |  | rs2119183 | rs2119183 |
|  |  | rs11503016 | rs11503016 |
|  |  | rs4695148 | rs4695148 |
|  |  | rs11503014 | rs11503014 |
|  |  | rs3756007 | rs3756007 |
| *SLC1A1* | Glutamate | rs7864496 | rs10117931, rs7872075, rs7864496, rs2228622, rs7871691, rs12551465, rs11792309, rs7858877, rs10974629, rs3780412, |
|  |  |  | rs7042333, rs7871243 |
|  |  | rs2150195 | rs13289148, rs7030825, rs1888279, rs10118909, rs1980943, rs2150195, rs10814997, rs2039291 |
|  |  | rs12342908 | rs10115600, rs10974587, rs12342908, rs2094806, rs9918970 |
|  |  | rs7022369 | rs7867651, rs7849913, rs7851538, rs7022369, rs7862687 |
|  |  | rs7022772 | rs3780414, rs3780413, rs6476878, rs4740790, rs7022772 |
|  |  | rs301979 | rs190978, rs188537, rs301979 |
|  |  | rs12378107 | rs11791930, rs12378107 |
|  |  | rs2039290 | rs2039290, rs2039216 |
|  |  | rs10739064 | rs10739065, rs10739064 |
|  |  | rs1471786 | rs301431, rs1471786 |
|  |  | rs4742004 | rs4742004, rs7021569 |
|  |  | rs10814998 | rs10491732, rs10814998, rs16921385 |
|  |  | rs12341219 | rs12341219, rs10815018 |
|  |  | rs10491735 | rs4740788, rs10491735 |
|  |  | rs2183472 | rs1360329, rs2183472 |
|  |  | rs10739066 | rs10739066, rs3780415 |
|  |  | rs10815016 | rs10815015, rs10815016 |
|  |  | rs4742003 | rs10815002, rs4742003 |
|  |  | rs10491731 | rs10974611 |
|  |  | rs7858819 | rs10815020, rs7858819 |
|  |  | rs10974591 | rs10974591 |
|  |  | rs10814991 | rs10814991 |
|  |  | rs12553697 | rs12553697 |
|  |  | rs6476873 | rs6476873 |
|  |  | rs10491734 | rs10491734 |
|  |  | rs7856675 | rs7856675 |
|  |  | rs6476879 | rs6476879 |
|  |  | rs7031998 | rs7031998 |
|  |  | rs16921457 | rs16921457 |
|  |  | rs10739062 | rs10739062 |
|  |  | rs928209 | rs928209 |
|  |  | rs301443 | rs301443 |
|  |  | rs10974616 | rs10974616 |
|  |  | rs9775228 | rs9775228 |
|  |  | rs10758632 | rs10758632 |
|  |  | rs7021409 | rs7021409 |
|  |  | rs184204 | rs184204 |
|  |  | rs10758624 | rs10758624 |
|  |  | rs10814993 | rs10814993 |
|  |  | rs10758631 | rs10758631 |
|  |  | rs301434 | rs301434 |
|  |  | rs301439 | rs301439 |
|  |  | rs17812372 | rs17812372 |
|  |  | rs972519 | rs972519 |
|  |  | rs10814988 | rs10814988 |
|  |  | rs7848533 | rs7848533 |
|  |  | rs2150192 | rs2150192 |
|  |  | rs10974620 | rs10974620 |
|  |  | rs10974619 | rs10974619 |
|  |  | rs301445 | rs301445 |
|  |  | rs10815013 | rs10815013 |
|  |  | rs10815019 | rs10815019 |
| **Newly investigated candidate gene** |  |  |  |
| *HRH3* | Histamine | rs944887 ^d^ | rs1614845, rs944887 |
|  |  | rs3787430 | rs3787430 |
|  |  | rs1760042 | rs1760042 |
|  |  | rs6587299 | rs6587299 |
|  |  | rs6062144 | rs6062144 |
|  |  | rs6061458 | rs6061458 |
|  |  | rs3787429 ^d^ | rs3787429 |
|  |  | rs6142998 | rs6142998 |

TS, Tourette syndrome; OCD, obsessive-compulsive disorder; ASD, autism spectrum disorder; tSNPs, tagging SNPs; *DRD2*, dopamine receptor D2; *HDC*, l-histidine decarboxylase; *MAO-A*, monoamine oxidase-A; *SLC6A3*, solute carrier family 6, dopamine transporter; *TPH2*, tryptophan hydroxylase 2; *GABRA2*, GABA-A receptor, alpha 2; *SLC1A1*, solute carrier family 1 member 1, glutamate transporter; *HRH3*, histamine receptor H3.

^a^Candidate genes were identified in a literature review; PubMed searches were performed in October 2014 and as such only available studies up to that date were evaluated. PubMed searches were conducted using the terms “Tourette”, “tics”, and “TS” in combination with the terms “candidate gene” and “association study”.

^b^The r^2^ threshold for the tSNP selection was set at 0.8 using the HapMap CEU population and Haploview [1–3]. Selection also included tSNPs 10 kb upstream and downstream of each gene.

^c^List of SNPs in linkage disequilibrium with the selected tSNP.

^d^tSNP did not pass standard quality control checks in PLINK V1.07 using the recommended parameters published in [4, 5].

Table S2: Number of parent-child trio’s genotyped per single nucleotide polymorphism^a^

| SNP | CHR | Gene | Array | N parent-child trios before quality control | N parent-child trios after quality control |
| --- | --- | --- | --- | --- | --- |
| rs11264126 | chr01 | DLGAP3 | GoldenGate and OmniExpressExome | 465 | 371 |
| rs13063502 | chr03 |  | GoldenGate and OmniExpressExome | 465 | 371 |
| rs1442060 | chr04 | GABRA2 | GoldenGate and OmniExpressExome | 465 | 371 |
| rs16859227 | chr04 | GABRA2 | GoldenGate and OmniExpressExome | 465 | 371 |
| rs3849591 | chr04 | GABRA2 | GoldenGate and OmniExpressExome | 465 | 371 |
| rs10064525 | chr05 | SLC6A3/DAT1 | GoldenGate and OmniExpressExome | 465 | 371 |
| rs1042098 | chr05 | SLC6A3/DAT1 | GoldenGate and OmniExpressExome | 465 | 371 |
| rs12516758 | chr05 | SLC6A3/DAT1 | GoldenGate and OmniExpressExome | 465 | 371 |
| rs27048 | chr05 | SLC6A3/DAT1 | GoldenGate and OmniExpressExome | 465 | 371 |
| rs40184 | chr05 | SLC6A3/DAT1 | GoldenGate and OmniExpressExome | 465 | 371 |
| rs6350 | chr05 | SLC6A3/DAT1 | GoldenGate and OmniExpressExome | 465 | 371 |
| rs6869645 | chr05 | SLC6A3/DAT1 | GoldenGate and OmniExpressExome | 465 | 371 |
| rs7711337 | chr05 |  | GoldenGate and OmniExpressExome | 465 | 371 |
| rs9357271 | chr06 | BTBD9 | GoldenGate and OmniExpressExome | 465 | 371 |
| rs7794745 | chr07 | CNTNAP2 | GoldenGate and OmniExpressExome | 465 | 371 |
| rs7834018 | chr08 |  | GoldenGate and OmniExpressExome | 465 | 371 |
| rs10491734 | chr09 | SLC1A1 | GoldenGate and OmniExpressExome | 465 | 371 |
| rs10758624 | chr09 | SLC1A1 | GoldenGate and OmniExpressExome | 465 | 371 |
| rs10758631 | chr09 | SLC1A1 | GoldenGate and OmniExpressExome | 465 | 371 |
| rs10815016 | chr09 | SLC1A1 | GoldenGate and OmniExpressExome | 465 | 371 |
| rs10974619 | chr09 | SLC1A1 | GoldenGate and OmniExpressExome | 465 | 371 |
| rs1471786 | chr09 | SLC1A1 | GoldenGate and OmniExpressExome | 465 | 371 |
| rs16921457 | chr09 | SLC1A1 | GoldenGate and OmniExpressExome | 465 | 371 |
| rs184204 | chr09 | SLC1A1 | GoldenGate and OmniExpressExome | 465 | 371 |
| rs2150192 | chr09 | SLC1A1 | GoldenGate and OmniExpressExome | 465 | 371 |
| rs2150195 | chr09 | SLC1A1 | GoldenGate and OmniExpressExome | 465 | 371 |
| rs2183472 | chr09 | SLC1A1 | GoldenGate and OmniExpressExome | 465 | 371 |
| rs4742004 | chr09 | SLC1A1 | GoldenGate and OmniExpressExome | 465 | 371 |
| rs6476879 | chr09 | SLC1A1 | GoldenGate and OmniExpressExome | 465 | 371 |
| rs7021409 | chr09 | SLC1A1 | GoldenGate and OmniExpressExome | 465 | 371 |
| rs7022772 | chr09 | SLC1A1 | GoldenGate and OmniExpressExome | 465 | 371 |
| rs7848533 | chr09 | SLC1A1 | GoldenGate and OmniExpressExome | 465 | 371 |
| rs7856675 | chr09 | SLC1A1 | GoldenGate and OmniExpressExome | 465 | 371 |
| rs7864496 | chr09 | SLC1A1 | GoldenGate and OmniExpressExome | 465 | 371 |
| rs7868992 | chr09 | COL27A1 | GoldenGate and OmniExpressExome | 465 | 371 |
| rs1079597 | chr11 | DRD2 | GoldenGate and OmniExpressExome | 465 | 371 |
| rs10891556 | chr11 | DRD2 | GoldenGate and OmniExpressExome | 465 | 371 |
| rs11607165 | chr11 | STIP1 | GoldenGate and OmniExpressExome | 465 | 371 |
| rs12364283 | chr11 | DRD2 | GoldenGate and OmniExpressExome | 465 | 371 |
| rs12422191 | chr11 | DRD2 | GoldenGate and OmniExpressExome | 465 | 371 |
| rs12574471 | chr11 | DRD2 | GoldenGate and OmniExpressExome | 465 | 371 |
| rs12575642 | chr11 | FERMT3 | GoldenGate and OmniExpressExome | 465 | 371 |
| rs17529477 | chr11 | DRD2 | GoldenGate and OmniExpressExome | 465 | 371 |
| rs4245148 | chr11 | DRD2 | GoldenGate and OmniExpressExome | 465 | 371 |
| rs4648318 | chr11 | DRD2 | GoldenGate and OmniExpressExome | 465 | 371 |
| rs7131056 | chr11 | DRD2 | GoldenGate and OmniExpressExome | 465 | 371 |
| rs10879357 | chr12 | TPH2 | GoldenGate and OmniExpressExome | 465 | 371 |
| rs11178993 | chr12 | TPH2 | GoldenGate and OmniExpressExome | 465 | 371 |
| rs11178999 | chr12 | TPH2 | GoldenGate and OmniExpressExome | 465 | 371 |
| rs12231341 | chr12 | TPH2 | GoldenGate and OmniExpressExome | 465 | 371 |
| rs1386488 | chr12 | TPH2 | GoldenGate and OmniExpressExome | 465 | 371 |
| rs1487275 | chr12 | TPH2 | GoldenGate and OmniExpressExome | 465 | 371 |
| rs1872824 | chr12 | TPH2 | GoldenGate and OmniExpressExome | 465 | 371 |
| rs297941 | chr12 |  | GoldenGate and OmniExpressExome | 465 | 371 |
| rs4565946 | chr12 | TPH2 | GoldenGate and OmniExpressExome | 465 | 371 |
| rs4570625 | chr12 | TPH2 | GoldenGate and OmniExpressExome | 465 | 371 |
| rs4760820 | chr12 | TPH2 | GoldenGate and OmniExpressExome | 465 | 371 |
| rs6539267 | chr12 | POLR3B | GoldenGate and OmniExpressExome | 465 | 371 |
| rs7969998 | chr12 | TPH2 | GoldenGate and OmniExpressExome | 465 | 371 |
| rs7336083 | chr13 |  | GoldenGate and OmniExpressExome | 465 | 371 |
| rs9593835 | chr13 | SLITRK1 | GoldenGate and OmniExpressExome | 465 | 371 |
| rs9652236 | chr13 |  | GoldenGate and OmniExpressExome | 465 | 371 |
| rs3959666 | chr15 | HDC | GoldenGate and OmniExpressExome | 465 | 371 |
| rs4150167 | chr16 | TAF1C | GoldenGate and OmniExpressExome | 465 | 371 |
| rs11081062 | chr18 | DLGAP1 | GoldenGate and OmniExpressExome | 465 | 371 |
| rs6131295 | chr20 |  | GoldenGate and OmniExpressExome | 465 | 371 |
| rs6587299 | chr20 | HRH3 | GoldenGate and OmniExpressExome | 465 | 371 |
| rs944887 | chr20 | HRH3 | GoldenGate and OmniExpressExome | 465 | 371 |
| rs4680 | chr22 | COMT | GoldenGate and OmniExpressExome | 465 | 371 |
| rs1181275 | chrX | MAO-A | GoldenGate and OmniExpressExome | 465 | 371 |
| rs3027409 | chrX | MAO-A | GoldenGate and OmniExpressExome | 465 | 371 |
| rs3027415 | chrX | MAO-A | GoldenGate and OmniExpressExome | 465 | 371 |
| rs3813929 | chrX | HTR2C | GoldenGate and OmniExpressExome | 465 | 371 |
| rs5906957 | chrX | MAO-A | GoldenGate and OmniExpressExome | 465 | 371 |
| rs6609257 | chrX | MAO-A | GoldenGate and OmniExpressExome | 465 | 371 |
| rs621942 | chr11 | PICALM | OmniExpressExome | 254 | 192 |
| rs7123010 | chr11 | ME3 | OmniExpressExome | 254 | 192 |
| rs11603305 | chr11 |  | OmniExpressExome | 254 | 192 |
| rs2060546 | chr12 |  | OmniExpressExome | 254 | 192 |
| rs12141243 | chr01 | DLGAP3 | GoldenGate | 211 | 179 |
| rs12037173 | chr01 | LRRC7 | GoldenGate | 211 | 179 |
| rs2556378 | chr02 | BCL11A | GoldenGate | 211 | 179 |
| rs4675502 | chr02 | PARD3B | GoldenGate | 211 | 179 |
| rs4988462 | chr03 | POU1F1 | GoldenGate | 211 | 179 |
| rs10013922 | chr04 | GABRA2 | GoldenGate | 211 | 179 |
| rs279828 | chr04 | GABRA2 | GoldenGate | 211 | 179 |
| rs1372472 | chr04 |  | GoldenGate | 211 | 179 |
| rs17537141 | chr04 | GABRA2 | GoldenGate | 211 | 179 |
| rs16859354 | chr04 | GABRA2 | GoldenGate | 211 | 179 |
| rs17537359 | chr04 | GABRA2 | GoldenGate | 211 | 179 |
| rs2119183 | chr04 | GABRA2 | GoldenGate | 211 | 179 |
| rs11503016 | chr04 | GABRA2 | GoldenGate | 211 | 179 |
| rs4695148 | chr04 | GABRA2 | GoldenGate | 211 | 179 |
| rs11503014 | chr04 | GABRA2 | GoldenGate | 211 | 179 |
| rs3756007 | chr04 | GABRA2 | GoldenGate | 211 | 179 |
| rs4307059 | chr05 |  | GoldenGate | 211 | 179 |
| rs13176113 | chr05 |  | GoldenGate | 211 | 179 |
| rs458860 | chr05 | SLC6A3 | GoldenGate | 211 | 179 |
| rs250686 | chr05 | SLC6A3 | GoldenGate | 211 | 179 |
| rs11564772 | chr05 | SLC6A3 | GoldenGate | 211 | 179 |
| rs11564758 | chr05 | SLC6A3 | GoldenGate | 211 | 179 |
| rs10052016 | chr05 | SLC6A3 | GoldenGate | 211 | 179 |
| rs2550948 | chr05 |  | GoldenGate | 211 | 179 |
| rs12654851 | chr05 |  | GoldenGate | 211 | 179 |
| rs6347 | chr05 | SLC6A3 | GoldenGate | 211 | 179 |
| rs2550956 | chr05 |  | GoldenGate | 211 | 179 |
| rs37022 | chr05 | SLC6A3 | GoldenGate | 211 | 179 |
| rs2617605 | chr05 | SLC6A3 | GoldenGate | 211 | 179 |
| rs2735917 | chr05 | SLC6A3 | GoldenGate | 211 | 179 |
| rs27074 | chr05 |  | GoldenGate | 211 | 179 |
| rs466630 | chr05 | SLC6A3 | GoldenGate | 211 | 179 |
| rs246993 | chr05 |  | GoldenGate | 211 | 179 |
| rs9499708 | chr06 |  | GoldenGate | 211 | 179 |
| rs1718101 | chr07 | CNTNAP2 | GoldenGate | 211 | 179 |
| rs769111 | chr07 |  | GoldenGate | 211 | 179 |
| rs12342908 | chr09 | SLC1A1 | GoldenGate | 211 | 179 |
| rs7022369 | chr09 | SLC1A1 | GoldenGate | 211 | 179 |
| rs301979 | chr09 | SLC1A1 | GoldenGate | 211 | 179 |
| rs12378107 | chr09 | SLC1A1 | GoldenGate | 211 | 179 |
| rs2039290 | chr09 | SLC1A1 | GoldenGate | 211 | 179 |
| rs10739064 | chr09 | SLC1A1 | GoldenGate | 211 | 179 |
| rs10814998 | chr09 | SLC1A1 | GoldenGate | 211 | 179 |
| rs12341219 | chr09 | SLC1A1 | GoldenGate | 211 | 179 |
| rs10491735 | chr09 |  | GoldenGate | 211 | 179 |
| rs10739066 | chr09 | SLC1A1 | GoldenGate | 211 | 179 |
| rs4742003 | chr09 | SLC1A1 | GoldenGate | 211 | 179 |
| rs10491731 | chr09 | SLC1A1 | GoldenGate | 211 | 179 |
| rs7858819 | chr09 | SLC1A1 | GoldenGate | 211 | 179 |
| rs10974591 | chr09 | SLC1A1 | GoldenGate | 211 | 179 |
| rs10814991 | chr09 | SLC1A1 | GoldenGate | 211 | 179 |
| rs12553697 | chr09 |  | GoldenGate | 211 | 179 |
| rs6476873 | chr09 | SLC1A1 | GoldenGate | 211 | 179 |
| rs7031998 | chr09 | SLC1A1 | GoldenGate | 211 | 179 |
| rs10739062 | chr09 | SLC1A1 | GoldenGate | 211 | 179 |
| rs928209 | chr09 | SLC1A1 | GoldenGate | 211 | 179 |
| rs301443 | chr09 |  | GoldenGate | 211 | 179 |
| rs10974616 | chr09 | SLC1A1 | GoldenGate | 211 | 179 |
| rs9775228 | chr09 |  | GoldenGate | 211 | 179 |
| rs10758632 | chr09 | SLC1A1 | GoldenGate | 211 | 179 |
| rs10814993 | chr09 | SLC1A1 | GoldenGate | 211 | 179 |
| rs301434 | chr09 | SLC1A1 | GoldenGate | 211 | 179 |
| rs301439 | chr09 |  | GoldenGate | 211 | 179 |
| rs17812372 | chr09 |  | GoldenGate | 211 | 179 |
| rs972519 | chr09 | SLC1A1 | GoldenGate | 211 | 179 |
| rs10814988 | chr09 |  | GoldenGate | 211 | 179 |
| rs10974620 | chr09 | SLC1A1 | GoldenGate | 211 | 179 |
| rs301445 | chr09 |  | GoldenGate | 211 | 179 |
| rs10815013 | chr09 | SLC1A1 | GoldenGate | 211 | 179 |
| rs10815019 | chr09 | SLC1A1 | GoldenGate | 211 | 179 |
| rs2587548 | chr11 | DRD2 | GoldenGate | 211 | 179 |
| rs2587550 | chr11 |  | GoldenGate | 211 | 179 |
| rs7122454 | chr11 | DRD2 | GoldenGate | 211 | 179 |
| rs17115583 | chr11 | DRD2 | GoldenGate | 211 | 179 |
| rs4245147 | chr11 | DRD2 | GoldenGate | 211 | 179 |
| rs2234689 | chr11 |  | GoldenGate | 211 | 179 |
| rs1800497 | chr11 | ANKK1 | GoldenGate | 211 | 179 |
| rs11214606 | chr11 | DRD2 | GoldenGate | 211 | 179 |
| rs6279 | chr11 | DRD2 | GoldenGate | 211 | 179 |
| rs5016282 | chr11 | GRM5 | GoldenGate | 211 | 179 |
| rs4271390 | chr11 | PVRL1 | GoldenGate | 211 | 179 |
| rs7955501 | chr12 | TPH2 | GoldenGate | 211 | 179 |
| rs1386497 | chr12 | TPH2 | GoldenGate | 211 | 179 |
| rs17110536 | chr12 | TPH2 | GoldenGate | 211 | 179 |
| rs10879358 | chr12 | TPH2 | GoldenGate | 211 | 179 |
| rs10748185 | chr12 | TPH2 | GoldenGate | 211 | 179 |
| rs17722134 | chr12 | TPH2 | GoldenGate | 211 | 179 |
| rs4760813 | chr12 |  | GoldenGate | 211 | 179 |
| rs17110747 | chr12 | TPH2 | GoldenGate | 211 | 179 |
| rs11615016 | chr12 | TPH2 | GoldenGate | 211 | 179 |
| rs12424836 | chr12 |  | GoldenGate | 211 | 179 |
| rs11149058 | chr13 |  | GoldenGate | 211 | 179 |
| rs9531520 | chr13 |  | GoldenGate | 211 | 179 |
| rs8029462 | chr15 | SLC27A2 | GoldenGate | 211 | 179 |
| rs11856059 | chr15 |  | GoldenGate | 211 | 179 |
| rs1365503 | chr15 |  | GoldenGate | 211 | 179 |
| rs854157 | chr15 |  | GoldenGate | 211 | 179 |
| rs2853766 | chr15 |  | GoldenGate | 211 | 179 |
| rs854158 | chr15 |  | GoldenGate | 211 | 179 |
| rs854159 | chr15 |  | GoldenGate | 211 | 179 |
| rs2070595 | chr15 |  | GoldenGate | 211 | 179 |
| rs8034597 | chr15 |  | GoldenGate | 211 | 179 |
| rs854150 | chr15 |  | GoldenGate | 211 | 179 |
| rs9920021 | chr15 |  | GoldenGate | 211 | 179 |
| rs662669 | chr17 | TBCD | GoldenGate | 211 | 179 |
| rs3744161 | chr17 | TBCD | GoldenGate | 211 | 179 |
| rs3787430 | chr20 | HRH3 | GoldenGate | 211 | 179 |
| rs1760042 | chr20 |  | GoldenGate | 211 | 179 |
| rs6062144 | chr20 |  | GoldenGate | 211 | 179 |
| rs6061458 | chr20 |  | GoldenGate | 211 | 179 |
| rs3787429 | chr20 | HRH3 | GoldenGate | 211 | 179 |
| rs6142998 | chr20 |  | GoldenGate | 211 | 179 |
| rs518147 | chrX | HTR2C | GoldenGate | 211 | 179 |
| rs5906729 | chrX | MAO-A | GoldenGate | 211 | 179 |
| rs5905859 | chrX | MAO-A | GoldenGate | 211 | 179 |
| rs3027399 | chrX | MAO-A | GoldenGate | 211 | 179 |
| rs12843533 | chrX | MAO-A | GoldenGate | 211 | 179 |

^a^GoldenGate: Illumina GoldenGate Genotyping Assay; OmniExpressExome: Illumina HumanOmniExpressExome v1.2 BeadChip array.

**Table S3:** Power calculations for parent-child trios at different sample sizes, odds ratios, and minor allele frequencies^a^

| parent-child trio’s^b^ | OR | Power (MAF = 0.05) | Power (MAF = 0.10) | Power (MAF = 0.20) | Power (MAF = 0.30) | Power (MAF = 0.40) |
| --- | --- | --- | --- | --- | --- | --- |
| N= 371 | **1.1** | 0.069 | 0.0868 | 0.115 | 0.136 | 0.147 |
|  | **1.2** | 0.125 | 0.193 | 0.302 | 0.373 | 0.410 |
|  | **1.3** | 0.214 | 0.356 | 0.553 | 0.658 | 0.704 |
|  | **1.4** | 0.328 | 0.542 | 0.773 | 0.862 | 0.893 |
|  | **1.5** | 0.454 | 0.711 | 0.908 | 0.958 | 0.971 |
|  | **1.6** | 0.581 | 0.837 | 0.969 | 0.990 | 0.994 |
|  | **1.7** | 0.694 | 0.918 | 0.992 | 0.998 | 0.999 |
|  | **1.8** | 0.787 | 0.963 | 0.998 | 0.999 | 0.999 |
|  | **1.9** | 0.859 | 0.984 | 0.999 | 0.999 | 0.999 |
|  | **2.0** | 0.910 | 0.994 | 0.999 | 0.999 | 0.999 |
| N= 192 | **1.1** | 0.059 | 0.069 | 0.083 | 0.094 | 0.099 |
|  | **1.2** | 0.088 | 0.123 | 0.179 | 0.217 | 0.238 |
|  | **1.3** | 0.133 | 0.208 | 0.325 | 0.399 | 0.435 |
|  | **1.4** | 0.192 | 0.318 | 0.495 | 0.592 | 0.635 |
|  | **1.5** | 0.264 | 0.440 | 0.657 | 0.756 | 0.794 |
|  | **1.6** | 0.343 | 0.563 | 0.788 | 0.869 | 0.896 |
|  | **1.7** | 0.427 | 0.674 | 0.879 | 0.937 | 0.953 |
|  | **1.8** | 0.510 | 0.769 | 0.936 | 0.972 | 0.980 |
|  | **1.9** | 0.589 | 0.841 | 0.968 | 0.989 | 0.992 |
|  | **2.0** | 0.661 | 0.895 | 0.985 | 0.995 | 0.999 |
| N= 179 | **1.1** | 0.059 | 0.067 | 0.081 | 0.091 | 0.096 |
|  | **1.2** | 0.086 | 0.118 | 0.170 | 0.206 | 0.225 |
|  | **1.3** | 0.127 | 0.197 | 0.307 | 0.376 | 0.411 |
|  | **1.4** | 0.183 | 0.299 | 0.468 | 0.563 | 0.605 |
|  | **1.5** | 0.249 | 0.415 | 0.627 | 0.727 | 0.765 |
|  | **1.6** | 0.324 | 0.534 | 0.759 | 0.846 | 0.875 |
|  | **1.7** | 0.403 | 0.644 | 0.856 | 0.921 | 0.934 |
|  | **1.8** | 0.482 | 0.738 | 0.920 | 0.963 | 0.973 |
|  | **1.9** | 0.559 | 0.815 | 0.958 | 0.983 | 0.988 |
|  | **2.0** | 0.631 | 0.874 | 0.979 | 0.993 | 0.995 |

OR, odds ratio; MAF, minor allele frequency.

^a^For all power calculations α was set at 0.05

^b^The sample sizes listed correspond to the full dataset (N = 371), SNPs only genotyped on the Illumina HumanOmniExpressExome v1.2 BeadChip array (N=192), and SNPs only genotyped on the Illumina GoldenGate Genotyping Assay (N= 179).

**Table S4:** Haplotype based transmission disequilibrium for *TPH2* SNPs rs4570625 and rs4565946 in TS

| Locus | Haplotype | T:U | χ^2^ | *P-value* |
| --- | --- | --- | --- | --- |
| rs4570625\|rs4565946 | GT | 197:161 | 3.62 | 0.05709 |
| rs4570625\|rs4565946 | TC | 119:130 | 0.4859 | 0.4857 |
| rs4570625\|rs4565946 | GC | 150:176 | 2.074 | 0.1499 |
| rs4570625\|rs4565946 | TT | 0:0 | - | - |

*TPH2*, tryptophan hydroxylase 2; TS, Tourette syndrome; T:U, transmitted:untransmitted count of haplotype

Table S5: Transmission disequilibrium tests results of available individual candidate SNPs previously implicated in TS and OCD after quality control check^a^

| **SNP ID** | **CHR** | **Gene** | **Minor allele** | **Minor allele frequency^b^** | **Major allele** | **Major allele frequency^b^** | **T:U** | **OR** | **95% CI OR** | **χ^2^** | ***P-value* nominal** | ***P-value* adjusted (FDR) ^c^** |
| --- | --- | --- | --- | --- | --- | --- | --- | --- | --- | --- | --- | --- |
| **Previously implicated in TS** |  |  |  |  |  |  |  |  |  |  |  |  |
| rs1800497 | 11 | *ANKK1/DRD2* | A | 0.18 | G | 0.82 | 68:74 | 1.09 | 0.78-1.51 | 0.14 | 0.73 | 0.82 |
| rs9357271 | 6 | *BTBD9* | C | 0.24 | T | 0.76 | 151:131 | 1.15 | 0.91-1.46 | 0.50 | 0.49 | 0.82 |
| rs11264126 | 1 | *DLGAP3* | A | 0.49 | G | 0.51 | 171:165 | 1.04 | 0.84-1.28 | 0.87 | 0.36 | 0.82 |
| rs12141243 | 1 | *DLGAP3* | C | 0.13 | T | 0.87 | 55:49 | 1.12 | 0.76-1.65 | 0.18 | 0.73 | 0.82 |
| rs6279 | 11 | *DRD2* | G | 0.31 | C | 0.69 | 89:79 | 1.13 | 0.83-1.53 | 0.31 | 0.64 | 0.82 |
| rs1079597 | 11 | *DRD2* | T | 0.11 | C | 0.89 | 90:100 | 1.11 | 0.84-1.48 | 0.52 | 0.49 | 0.82 |
| rs4648318 | 11 | *DRD2* | C | 0.25 | T | 0.75 | 133:121 | 1.10 | 0.86-1.41 | 0.55 | 0.50 | 0.82 |
| rs854150 | 15 | *HDC* | G | 0.36 | C | 0.64 | 102:97 | 1.05 | 0.80-1.39 | 0.37 | 0.53 | 0.82 |
| rs518147 | 23 | *HTR2C* | C | 0.37 | G | 0.63 | 51:41 | 1.24 | 0.82-1.88 | 0.41 | 0.56 | 0.82 |
| rs3813929 | 23 | *HTR2C* | T | 0.18 | C | 0.82 | 43:42 | 1.02 | 0.67-1.57 | 0.03 | 0.90 | 0.90 |
| rs6347 | 5 | *SLC6A3/DAT1* | C | 0.24 | T | 0.76 | 79:76 | 1.04 | 0.76-1.42 | 0.17 | 0.74 | 0.82 |
| rs9593835 | 13 | *SLITRK1* | C | 0.26 | T | 0.74 | 132:149 | 1.13 | 0.89-1.43 | 0.11 | 0.77 | 0.82 |
| rs9531520 | 13 | *SLITRK1* | T | 0.19 | C | 0.81 | 46:67 | 1.46 | 1.00-2.12 | 1.29 | 0.32 | 0.82 |
| rs3744161 | 17 | *TBCD* | G | 0.46 | A | 0.54 | 123:91 | 1.35 | 1.03-1.77 | 5.45 | 0.03 | 0.26 |
| rs662669 | 17 | *TBCD* | C | 0.42 | T | 0.58 | 91:115 | 1.26 | 0.96-1.66 | 2.34 | 0.15 | 0.82 |
| rs4565946 | 12 | *TPH2* | T | 0.49 | C | 0.51 | 205:163 | 1.26 | 1.02-1.55 | 6.01 | 0.02 | 0.26 |
| rs4570625 | 12 | *TPH2* | T | 0.20 | G | 0.80 | 124:134 | 1.08 | 0.85-1.38 | 0.21 | 0.67 | 0.82 |
| **Implicated in related disorder** |  |  |  |  |  |  |  |  |  |  |  |  |
| rs4680 | 22 | *COMT* | A | 0.47 | G | 0.53 | 116:128 | 1.10 | 0.86-1.42 | 0.48 | 0.48 | 0.82 |
| rs7794745 | 7 | *CNTNAP2* | T | 0.31 | A | 0.69 | 150:146 | 1.03 | 0.82-1.29 | 0.10 | 0.77 | 0.82 |

TS, Tourette syndrome; OCD, obsessive-compulsive disorder; CHR, chromosome; T:U, transmitted:untransmitted count for the minor allele; OR, odds ratio; 95% CI, 95% confidence interval; FDR, false discovery rate; *ANKK1*, Ankyrin Repeat And Kinase Domain Containing 1; *DRD2*, dopamine receptor D2; *BTBD9*, BTB (POZ) Domain Containing 9; *CNTNAP2*, Contactin Associated Protein-Like 2; *DLGAP3*, Discs, Large (Drosophila) Homolog-Associated Protein 3; TDT, transmission disequilibrium test; *HDC*, l-histidine decarboxylase; 5-HT receptor 2C; *SLC6A3*, solute carrier family 6, dopamine transporter; *DAT1*, Dopamine Transporter 1; *SLITRK1*, SLIT And NTRK-Like Family, Member 1; *TBCD*, Tubulin Folding Cofactor; *TPH2*, tryptophan hydroxylase 2; *COMT*, Catechol-O-Methyltransferase.

^a^All SNPs passed standard quality control checks in PLINK V1.07 using the recommended parameters published in [4, 5].

^b^Minor allele and major frequency were based on the HapMap-CEU population [1].

^c^FDR adjustment is only done for the number of SNPs listed in this table that passed quality control checks.

Table S6: Transmission disequilibrium tests results of candidate genes implicated in TS, OCD, and ASD

| **TS neurotransmitter-related candidate genes** | **CHR** | **χ^2^** | ***P-value* nominal** | ***P-value* adjusted (FDR)** **^a^** |
| --- | --- | --- | --- | --- |
| *DRD2* | 11 | 0.00 | 1.00 | 1.00 |
| *HDC* | 15 | 0.00 | 1.00 | 1.00 |
| *MAO-A* | X | 4.56 | 0.20 | 0.53 |
| *SLC6A3/DAT1* | 5 | 4.96 | 0.33 | 0.65 |
| *TPH2* | 12 | 6.24 | 0.12 | 0.47 |
| **Comorbidity-related candidate genes** |  |  |  |  |
| *GABRA2* | 4 | 0.00 | 1.00 | 1.00 |
| *SLC1A1* | 9 | 8.29 | 0.02 | 0.17 |
| **Newly investigated candidate gene** |  |  |  |  |
| *HRH3* | 20 | 0.00 | 1.00 | 1.00 |

TS, Tourette syndrome; OCD, obsessive-compulsive disorder; ASD, autism spectrum disorder; CHR, chromosome; FDR, false discovery rate; *DRD2*, dopamine receptor D2; *HDC*, l-histidine decarboxylase; *MAO-A*, monoamine oxidase-A; *SLC6A3/DAT1*, solute carrier family 6 / dopamine transporter; *TPH2*, tryptophan hydroxylase 2; *GABRA2*, GABA-A receptor, alpha 2; *SLC1A1*, solute carrier family 1 member 1, glutamate transporter; *HRH3*, histamine receptor H3.

^a^FDR adjustment is only done for the number of genes listed in this table.

Table S7: Transmission disequilibrium tests results of top SNPs implicated in GWAS of TS, OCD, ADHD, and ASD

| **TS GWAS SNPs** | **CHR** | **Gene name** | **Minor allele** | **Minor allele frequency** **^a^** | **Major allele ^a^** | **Major allele frequency** | **T:U** | **OR** | **95% CI** | **χ^2^** | ***P-value* nominal** | ***P-value* adjusted (FDR)** |
| --- | --- | --- | --- | --- | --- | --- | --- | --- | --- | --- | --- | --- |
| rs7868992 | 9 | *COL27A1* | G | 0.28 | A | 0.72 | 148:139 | 1.07 | 0.84-1.34 | 0.00 | 0.98 | 1.00 |
| rs621942 | 11 | *PICALM* | A | 0.24 | C | 0.76 | 121:84 | 1.44 | 1.09-1.90 | 7.08 | 0.01 | 0.24 |
| rs6539267 | 12 | *POLR3B* | C | 0.27 | T | 0.73 | 137:147 | 1.07 | 0.85-1.35 | 0.10 | 0.78 | 0.99 |
| rs4988462 | 3 | *POU1F1* | T | 0.44 | C | 0.56 | 91:110 | 1.21 | 0.67-1.16 | 0.09 | 0.80 | 0.99 |
| rs7123010 | 11 | *ME3* | A | 0.27 | G | 0.73 | 105:118 | 1.12 | 0.86-1.46 | 0.06 | 0.86 | 0.99 |
| rs2060546 ^b^ | 12 |  | A | 0.01 | G | 0.99 |  |  |  |  |  |  |
| rs13063502 | 3 |  | T | 0.18 | C | 0.82 | 91:71 | 1.28 | 0.94-1.75 | 1.31 | 0.27 | 0.95 |
| rs769111 | 7 |  | G | 0.37 | T | 0.63 | 95:87 | 1.09 | 0.82-1.46 | 0.99 | 0.36 | 0.99 |
| rs7336083 | 13 |  | A | 0.33 | G | 0.67 | 158:161 | 1.02 | 0.82-1.27 | 0.04 | 0.87 | 0.99 |
| rs11603305 | 11 |  | G | 0.32 | A | 0.68 | 125:92 | 1.36 | 1.04-1.78 | 6.11 | 0.02 | 0.24 |
| rs11149058 | 13 |  | C | 0.22 | T | 0.78 | 82:68 | 1.21 | 0.87-1.66 | 0.50 | 0.51 | 0.99 |
| rs4271390 ^b^ | 11 |  | C | 0.22 | T | 0.78 |  |  |  |  |  |  |
| **OCD GWAS SNPs** |  |  |  |  |  |  |  |  |  |  |  |  |
| rs11081062 | 18 | *DLGAP1* | T | 0.19 | C | 0.81 | 152:126 | 1.21 | 0.95-1.53 | 1.22 | 0.31 | 0.95 |
| rs9499708 | 6 |  | C | 0.42 | T | 0.58 | 171:155 | 1.10 | 0.89-1.37 | 0.36 | 0.57 | 0.99 |
| rs9652236 | 13 |  | T | 0.15 | G | 0.85 | 116:114 | 1.02 | 0.79-1.32 | 0.24 | 0.63 | 0.99 |
| rs6131295 | 20 |  | G | 0.23 | A | 0.77 | 134:144 | 1.07 | 0.85-1.36 | 0.19 | 0.68 | 0.99 |
| rs297941 ^b^ | 12 |  | G | 0.44 | A | 0.56 |  |  |  |  |  |  |
| **ADHD GWAS SNPs** |  |  |  |  |  |  |  |  |  |  |  |  |
| rs2556378 | 2 | *BCL11A* | T | 0.18 | G | 0.82 | 66:56 | 1.18 | 0.83-1.68 | 0.29 | 0.59 | 0.99 |
| rs12575642 | 11 | *FERMT3* | T | 0.15 | G | 0.85 | 112:86 | 1.30 | 0.98-1.73 | 1.93 | 0.19 | 0.95 |
| rs5016282 | 11 | *GRM5* | G | 0.15 | A | 0.85 | 1:8 | 8.00 | 1.00-63.98 | 3.27 | 0.09 | 0.53 |
| rs12037173 | 1 | *LRRC7* | G | 0.07 | A | 0.93 | 25:14 | 1.79 | 0.93-3.44 | 3.60 | 0.07 | 0.53 |
| rs11607165 | 11 | *STIP1* | G | 0.15 | T | 0.85 | 112:86 | 1.30 | 0.98-1.73 | 1.42 | 0.28 | 0.95 |
| **ASD GWAS SNPs** |  |  |  |  |  |  |  |  |  |  |  |  |
| rs1718101 | 7 | *CNTNAP2* | T | 0.07 | C | 0.93 | 25:28 | 1.12 | 0.65-1.92 | 0.01 | 1.00 | 1.00 |
| rs4675502 | 2 | *PARD3B* | G | 0.37 | A | 0.63 | 104:109 | 1.05 | 0.80-1.37 | 0.05 | 0.83 | 0.99 |
| rs4150167 ^b^ | 16 | *TAF1C* | T | 0.04 | C | 0.96 |  |  |  |  |  |  |
| rs4307059 | 5 |  | C | 0.37 | T | 0.63 | 100:113 | 1.13 | 0.86-1.48 | 0.71 | 0.45 | 0.99 |
| rs13176113 ^c^ | 5 |  | A | 0.28 | G | 0.72 | 99:113 | 1.14 | 0.87-1.49 | 0.61 | 0.47 | 0.99 |
| rs7834018 | 8 |  | C | 0.10 | T | 0.90 | 81:86 | 1.06 | 0.78-1.44 | 0.02 | 0.91 | 0.99 |
| rs7711337 | 5 |  | A | 0.40 | G | 0.60 | 171:179 | 1.05 | 0.85-1.29 | 0.09 | 0.79 | 0.99 |

TS, Tourette syndrome; GWAS, genome-wide association study; OCD, obsessive-compulsive disorder; ADHD, attention–deficit/hyperactivity disorder; ASD, autism spectrum disorder; CHR, chromosome; T:U, transmitted:untransmitted count for the minor allele; OR, odds ratio; 95% CI, 95% confidence interval; FDR, false discovery rate; *COL27A1*, Collagen, Type XXVII, Alpha 1*; PICALM*, Phosphatidylinositol Binding Clathrin Assembly Protein*; POLR3B*, Polymerase (RNA) III (DNA Directed) Polypeptide B*; POU1F1*, POU Class 1 Homeobox 1*; ME3*, Malic Enzyme 3*; DLGAP1*, Discs, Large (Drosophila) Homolog-Associated Protein 1*; BCL11A*, B-Cell CLL/Lymphoma 11A*; FERMT3*, Fermitin Family Member 3*; GRM5*, Glutamate Receptor, Metabotropic 5*; LRRC7*, Leucine Rich Repeat Containing 7*; STIP1*, Stress-Induced Phosphoprotein 1; *CNTNAP2*, Contactin Associated Protein-Like 2 *;PARD3B*, Par-3 Family Cell Polarity Regulator Beta; *TAF1C*, TATA Box Binding Protein (TBP)-Associated Factor.

^a^Minor allele and major frequency were based on the HapMap-CEU population [1].

^b^SNP did not pass standard quality control checks in PLINK V1.07 using the recommended parameters published in [4, 5].

^c^Original GWAS reported results for rs7704909 that is in high LD (R^2^=1) with rs13176113.

**References**

1 International T, Consortium H. The International HapMap Project. *Nature* 2003;**426**:789–96.

2 Barrett JC, Fry B, Maller J, Daly MJ. Haploview: Analysis and visualization of LD and haplotype maps. *Bioinformatics* 2005;**21**:263–5.

3 de Bakker PIW, Yelensky R, Pe’er I, Gabriel SB, Daly MJ, Altshuler D. Efficiency and power in genetic association studies. *Nat Genet* 2005;**37**:1217–23.

4 Purcell S, Neale B, Todd-Brown K, Thomas L, Ferreira MAR, Bender D, Maller J, Sklar P, de Bakker PIW, Daly MJ, Sham PC. PLINK: a tool set for whole-genome association and population-based linkage analyses. *Am J Hum Genet* 2007;**81**:559–75.

5 Anderson C a, Pettersson FH, Clarke GM, Cardon LR, Morris AP, Zondervan KT. Data quality control in genetic case-control association studies. *Nat Protoc* 2010;**5**:1564–73.
